# In vivo MR-angiography for the assessment of aortic aneurysms in an experimental mouse model on a clinical MRI scanner: Comparison with high-frequency ultrasound and histology

**DOI:** 10.1371/journal.pone.0178682

**Published:** 2017-06-05

**Authors:** Christian H. P. Jansen, Carolin Reimann, Julia Brangsch, René M. Botnar, Marcus R. Makowski

**Affiliations:** 1 King’s College London, Division of Imaging Sciences and Biomedical Engineering, London, United Kingdom; 2 Department of Radiology, Charite, Berlin, Germany; 3 BHF Centre of Excellence, King’s College London, London, United Kingdom; 4 Wellcome Trust and EPSRC Medical Engineering Center, King’s College London, London, United Kingdom; 5 NIHR Biomedical Research Centre, King’s College London, London, United Kingdom; 6 School of Engineering, Pontificia Universidad Catolica de Chile, Santiago, Chile; Universita degli Studi di Bologna, ITALY

## Abstract

**Background:**

MR-angiography currently represents one of the clinical reference-standards for the assessment of aortic-dimensions. For experimental research in mice, dedicated preclinical high-field MRI scanners are used in most studies. This type of MRI scanner is not available in most institutions. The aim of this study was to evaluate the potential of MR-angiography performed on a clinical MR scanner for the assessment of aortic aneurysms in an experimental mouse model, compared to a preclinical high-resolution ultrasound imaging system and histopathology.

**Methods:**

All *in vivo* MR imaging was performed with a clinical 3T MRI system (Philips Achieva) equipped with a clinical gradient system in combination with a single-loop surface-coil (47 mm). All MR sequences were based on clinically used sequences. For ultrasound, a dedicated preclinical high-resolution system (30 MHz linear transducer, Vevo770, VisualSonics) was used. All imaging was performed with an ApoE knockout mouse-model for aortic aneurysms. Histopathology was performed as reference-standard at all stages of aneurysm development.

**Results:**

MR-angiography on a clinical 3T system enabled the clear visualization of the aortic lumen and aneurysmal dilation at different stages of aneurysm development. A close correlation (R^2^ = 0.98; p < 0.001) with histological area measurements was found. Additionally, a good agreement between MR and ultrasound area measurements in systole (R^2^ = 0.91; p < 0.001) and diastole (R^2^ = 0.94; p < 0.001) were measured. Regarding interobserver reproducibility, MRI measurements yielded a smaller 95% confidence interval and a closer interreader correlation compared to ultrasound measurements (-0.37–0.46; R^2^ = 0.97 vs. -0.78–0.88; R^2^ = 0.87).

**Conclusion:**

This study demonstrates that MR-angiography, performed on a clinical 3T MR scanner, enables the reliable detection and quantification of the aortic dilatation at different stages of aneurysm development in an experimental mouse model.

## Introduction

Cardiovascular diseases, including aortic aneurysms, currently represent the main cause of death in Western societies. Especially the incidence of abdominal aortic aneurysms (AAAs) is steadily increasing, especially in the last 20 years [1, 2]. One of the main factors for this increase in incidence is the progressive aging of the general population. Currently the incidence of abdominal aortic aneurysms is estimated to be around 5% in the general population older than 50 years [3, 4]. The development of abdominal aortic aneurysms is associated with different causes, which include aortic infection, disorders of connective tissues and traumatic events [5, 6]. However, in most cases, the exact initiating event and pathophysiology, underlying the development is not fully understood yet [7]. In most cases, abdominal aortic aneurysms are associated with a progressive dilation of the aortic lumen. If this process continues, abdominal aortic rupture with fatal consequences can be the result [8].

In clinical practice, the screening for and the evaluation of abdominal aortic aneurysms can be performed using different imaging modalities [9]. These imaging modalities include magnetic resonance imaging (MRI), computed tomography (CT) and ultrasound (US). Each imaging technique is associated with specific advantages and disadvantages. MRI is unique in a sense that it enables the high-resolution 3D visualization of the aorta without the need for contrast agent or ionizing radiation. The main disadvantage of MRI is the relatively long scan time, compared to e.g. CT. The main advantage of CT is that imaging can be performed with a relatively high spatial resolution in a short time, however CT angiography is dependent on the use of iodinated contrast agents. Ultrasound has the advantage, that it is a widely distributed imaging technique available in most clinical centers. One of its main disadvantages is the operator dependence, which is especially relevant in the context of follow up examinations.

In magnetic resonance imaging, different techniques can be used for the visualization of vessels [9]. As MR contrast agents have been recently linked to side effects such as nephrogenic systemic fibrosis (NSF), non-contrast enhanced techniques are gaining in popularity [10]. In an experimental setting with small animals, such as mice, non-contrast enhanced techniques have several advantages. The main advantage is that imaging can be repeated longitudinally at limitless timepoints in a single animal without the need for intervention.

Experimental mouse models are the most widely used animal models for the preclinical investigation of diseases [11]. These models enable researchers to investigate the development of diseases and the influence of genetic modulation or pharmacological therapies on disease development. Additionally, most novel drugs are initially tested and validated in animal models prior to clinical trials. For most preclinical studies, histological analysis is performed to evaluate and quantify *in vivo* changes. However, noninvasive imaging techniques are gaining in importance. The main advantage of such an approach is that the number of required animals for a study is dramatically reduced.

In this context morphological as well as molecular imaging methods, including PET (positron emission tomography), SPECT (single-photon emission computed tomography), MRI, CT and ultrasound are the most frequently used techniques. In this group of modalities, MRI has several advantages including a unique soft tissue contrast in combination with the 3D acquisition of morphology and function [12, 13]. The majority of MRI studies in mice are performed with dedicated preclinical scanners with an ultra-high field strength (4.7–16.4 Tesla). Many institutions do not have these kind of preclinical imaging systems available as it usually requires dedicated personnel, including MR physicists, to run and maintain these systems. Advantages in hardware development and sequence design have made it possible to perform small animal imaging in clinical MRI scanners.

In this study, we evaluated the potential of a widely available clinical 3T the MRI system for the assessment of aortic aneurysm development in a mouse model. We used the most frequently investigated and best validated mouse model, which is based on the ApoE-/- mouse in combination with angiotensin II infusion [14–17]. Such a mouse model is highly relevant as abdominal aortic aneurysms represent a cardiovascular disease with severe complications. Many aspects of this experimental model are comparable to human disease including an increased incidence of hyperlipidemia [14]. Besides hyperlipidemia, other factors such as hypertension and cystic necrosis of the aortic wall also play an important role during the development of aortic aneurysms. Especially in the context of thoracic aortic aneurysms, a potential association with cystic medial necrosis has been described by previous studies [18, 19].

The aim of this study was to test the potential and reliability of a clinical 3T MRI system for the performance of MR-angiography in a mouse model of aortic aneurysm, compared to a dedicated preclinical high resolution ultrasound imaging system. Histological analysis was used as reference standard.

## Methods

### Setup of animal experiments

This study was carried out in strict accordance with the recommendations in the Guide for the Care and Use of Laboratory Animals of the United Kingdom Home Office and is regulated under the Animals Scientific Procedures Act 1986 (ASPA). ASPA has recently been revised to transpose to the European Directive 2010/63/EU on the protection of animals used for scientific purposes. The protocol was approved by the Committee on the Ethics of Animal Experiments of the King’s College London. All animal experiments in this study were performed in accordance with these international regulations. All intervention was performed with a combination anesthesia (Medetomidin, Midazolam, Fentanyl), and all efforts were made to minimize suffering. Osmotic minipumps (Alzet model 2004, Durect Corporation, Cupertino, CA, USA) were implanted into eight weeks old mice. For this study, homozygous eight weeks old C57BL/6J ApoE-knockout mice (male) from the Charles Rivers Laboratories were used. The animals were fed with a standard lab diet and housed in a clean barrier. The minipumps were loaded (loading was performed as suggested by the manufacturer) ex vivo with AngII (Sigma-Aldrich, Saint Louis, MO, USA,), implanted subcutaneously in the dorsal region under a combination anesthesia (500 μg/kg Medetomidin, 50 μg/kg Fentanyl, 5 mg/kg Midazolam) and infused a continuously dose of 1 microgram kg^-1^ min^-1^ into the mice [15, 20]. At week one, two, three and four after AngII infusion MRI imaging was performed and vessels were harvested for histological analysis each week (n = 8 per group). A sham-operated group (control group, n = 6) were also implanted minipumps which infused saline for four weeks. Eight mice were scanned by MRI at each time point. all animals were sacrificed for further histopathological analysis after the final imaging sessions. For the imaging session, mice were anesthetized with an intramuscular application [21, 22] of the same combination of Medetomidin, Fentanyl, Midazolam as mentioned above. In all mice with abdominal aortic aneurysm, an exsanguination in anterior perfusion with phosphate buffered saline (100 mm Hg) was performed following the MR imaging session. This was followed by a perfusion with 10% formalin if vessel samples were used for histology. Aorta, right renal artery and the last pair of intercostal arteries was excised to allow anatomical matching during histopathological processing of the samples.

### Animal handling and in vivo magnetic resonance imaging

**Fig 1:**
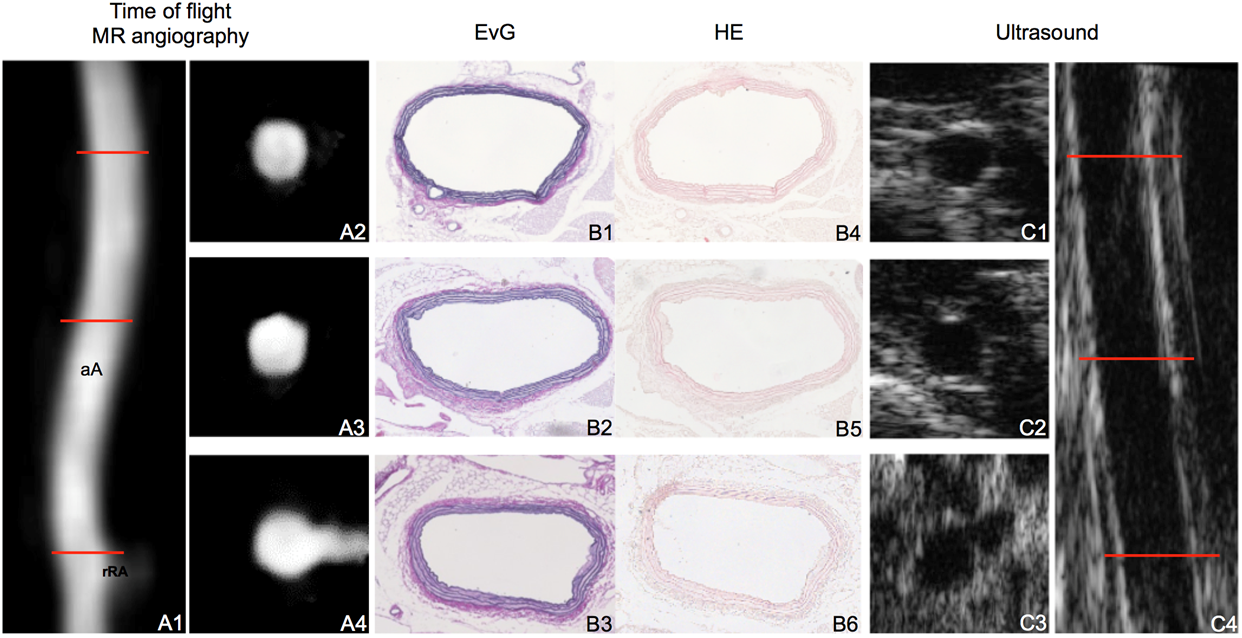
Visualization of the abdominal aorta in a control mouse by MR-angiography on a clinical MR system in comparison to high-frequency ultrasound. Images demonstrate the visualization of the abdominal aorta in a sham-operated ApoE-/- mouse by *in vivo* MR-angiography on a clinical MR system (A) and dedicated high-frequency ultrasound (C) in comparison to histology (B). The reconstructed TOF angiogram (A1, maximum intensity projection (MIP)) of the suprarenal part of the nondilated abdominal aorta is shown. Red lines indicate the orientation of subsequently performed transverse MRI sequences (A2, A3, A4). Corresponding *ex vivo* histological sections (B1- B6), Elastica van Gieson (EvG) stain (B1, B2, B3), hematoxylin eosin (HE) stain (B4, B5, B6) demonstrate a nondilated abdominal aorta at different levels (red lines). High-frequency ultrasound images (C) of an abdominal aorta using a dedicated imaging system (Vevo 770). Corresponding longitudinal (C4) and transversal imaging planes (C1, C2, C3) are shown.

Each animal was in anesthesia (as described earlier) and placed in a prone position on a surface microscopy coil (Philips healthcare, Best, the Netherlands). All imaging was performed using a clinical 3T Achieva MR system (Philips Healthcare, Best, The Netherlands). A gradient system with a gradient strength of 30 mT/m and a slew rate of 200 T/m/s was used. For the sequence acquisition, a dedicated software package for cardiac imaging was available. The signal was gained using a microscopy single loop coil with an inner diameter of 47 mm. The coil was placed in the magnetic center of the bore of the MRI. An MR compatible body temperature monitoring and heating system was used to maintain the temperature (37º degrees Celsius) of all animals during the entire acquisition of the MR data sets (Model 1025, SA Instruments Inc., Stony Brook, NY). All MR imaging sequences were based on clinical MR sequences. The imaging protocol included the following sequences. At the beginning of a MR imaging protocol a low-resolution scout sequence (three-dimensional gradient echo sequence) was used to for an anatomical overview and localization of the abdominal aorta. The scout scan was performed in the coronal and transverse orientation using the following parameters: field-of-view (FOV) = 200 mm, matrix = 320, slice thickness = 2 mm, TR/TE = 20/5.8ms, flip angle = 30° and slices = 9. Following a transverse orientation of a two-dimensional time-of-flight angiography (2D TOF) was executed for a precise visualization of the abdominal aorta. The 2D time-of-flight sequence was planned to include the renal arteries as anatomical landmarks in all scans. The image parameters included: Slice thickness = 0.5 mm, inplane spatial resolution = 0.3 x 0.3 mm (reconstructed 0.13 x 0.13 mm), imaging matrix = 160 x 160, field of view = 20 x 20 x 10 mm, flip angle = 60°, echo time (TE) 7.7 ms and repetition time (TR) sequence = 37 ms. Fold-over suppression was activated. Fold-over direction was right to left. A cartesian acquisition mode was used. A maximum intensity projection (MIP) in a 360-degree reconstruction was automatically reconstructed based on the time-of-flight angiography (Fig 1).

### Magnetic resonance image analysis

**Fig 2:**
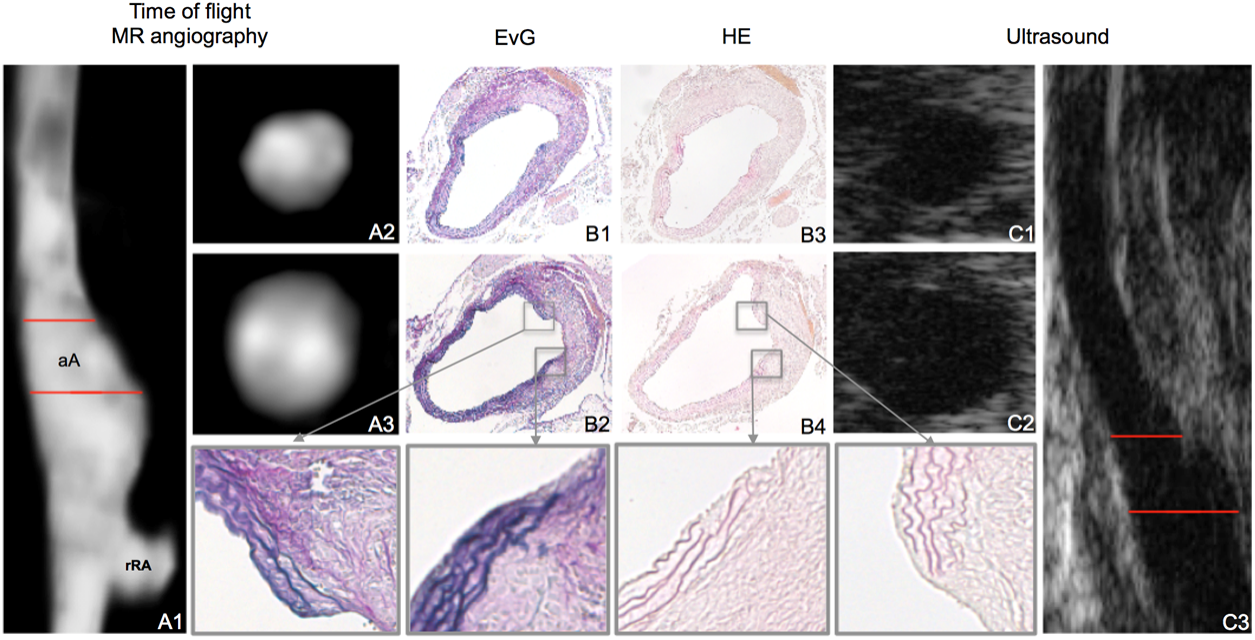
Evaluation of the abdominal aorta in an ApoE-/- mouse by MR-angiography on a clinical MR system and high-frequency ultrasound. Evaluation of an abdominal aortic aneurysm in an ApoE-/- mouse by *in vivo* MRI and ultrasound 4 week after continuous infusion of angiotensin II (4-week group). The maximum intensity projection (MIP) of the time-of-flight (TOF) angiogram (A1) demonstrates a significantly dilated aortic lumen. The location of transverse slices (A2, A3) are depicted by the red lines in A1. Corresponding *ex vivo* histological sections (Elastica van Gieson (EvG) stain (B1, B2), hematoxylin eosin (HE) stain (B3, B4) confirm the dilation of the aortic lumen. Magnifications of B2 and B4 highlight the site of rupture of the elastic laminae in the tunica media of the aorta in EvG stain and HE stain. Corresponding ultrasound images of abdominal aorta using the dedicated high-frequency US imaging system (Vevo 770) in sagittal (C3) and transversal orientation (C1, C2).

Signal to noise measurements (SNR) were performed directly proximal to the right renal artery in the control group (sham group, Fig 1). The right renal artery was clearly visible in all MR scans. If an aneurysm was present, signal to noise measurements (SNR) were performed at the location of the maximal area size (Fig 2). MR image analysis of DICOM images was performed using the open source version of OsiriX (version 7.1, OsiriX foundation). Time of flight (TOF) images were used to localize aortic aneurysms. Region of interests (ROIs) were measured as areas of signal enhancement on TOF images for the evaluation of signal intensity. Region of interests were drawn to delineate the complete vascular lumen for a reproducible measurement. For the ROIs signal to noise ratio (SNR) was calculated with following formula: Signal to noise ratio MR-angiography (MRA) = ((aneurysmal) aortic lumen signal) / (standard deviation lumen signal). Such an approach was chosen to allow for comparability between the analysis methods for MR and ultrasound.

### In vivo high-frequency ultrasound imaging

High resolution ultrasound imaging was performed with animals placed on a automatically heated table (37°) in supine position. Prior to ultrasound imaging, all animals were depilated with hair removal cream. For ultrasound measurements, a Vevo 770 ultrasound imaging system (VisualSonics, Toronto, Canada) with a 30 MHz linear signal transducer was used. For imaging and anatomical colocalization the suprarenal abdominal aorta more than 20 dynamic 2D-transverse and sagittal images were acquired in all animals. Cine transversal and sagittal images were reconstructed by the ECG-based kilohertz visualization (EKV) technique. The resulting images were analyzed during end-diastole and during end-systole.

### Analysis of in vivo high-frequency ultrasound imaging

Signal to noise measurements (SNR) were performed in the same animal at the same location as MR-angiography measurements. If an aneurysm was present, measurements were performed at the location of the maximal area size. If no aneurysm was present, measurements were performed directly proximal to the right renal artery. To obtain comparable measurements between MRI and ultrasound data sets comparable techniques for the assessment of the SNR were applied. Region of interests (ROIs) were measured as areas of signal enhancement of luminal aortic signal. The region of interest were drawn to delineate the complete vessel. For the ROIs signal to noise ratio (SNR) was calculated with following formula: Signal to noise ratio ultrasound (US) = ((aneurysmal) aortic lumen signal) / (standard deviation lumen signal).

### Aortic aneurysm morphometry

Aorta, right renal artery and the last pair of intercostal arteries was excised to allow precise anatomical matching between MRI, ultrasound and histopathology. The left renal artery and the last pair of intercostal artery were the main landmarks for the co-registration. The morphometrical analysis was performed using elastin-stained sections (Miller’s Elastica van Gieson stain) and ImageProPlus software (ImageProPlus, MediaCybernetics).

### Histological analysis of aortic aneurysms

Histological analysis was performed in the same region of aorta that was imaged with magnetic resonance imaging and ultrasound. *In vivo* and *ex vivo* morphometric data could therefore be directly compared. Surgically removed aortic aneurysms were processed overnight for further histological processing. Segmented aortic aneurysms were embedded in paraffin and were cut from the proximal end of the aneurysm every 40 μm into 6 μm thick serial sections. After dewaxing and rehydration, the sections were stained using Miller’s Elastica van Gieson stain (EvG) and hematoxylin and eosin (HE).

### Interobserver agreements magnetic resonance angiography and ultrasound measurements

For the assessment of the interreader variability two investigators performed the aortic measurements. All images were analyzed independently in a randomized order and blinded to the according other imaging modalities. Area sizes were recorded for each measurement.

### Statistical analysis

Data are expressed as mean ± standard deviation. A Student’s t test (two–tailed, unpaired) was used to compare continuous variables and verify the statistical significance between sham (control) and treated aortas. If more than two groups were investigated, a variance analysis (ANOVA) and Bonferroni correction was performed for statistical comparison. Interobserver agreements for *ex vivo* and *in vivo* measurements were assessed using Bland-Altman plots, which were generated for the raw volume data to display the spread of data and the limits of agreement. Linear regression was applied to determine the relationship between are measurements on MRI, ultrasound and histology.

## Results

### Assessment of different stages of abdominal aortic aneurysms by magnetic resonance angiography on a clinical MR system

**Fig 3:**
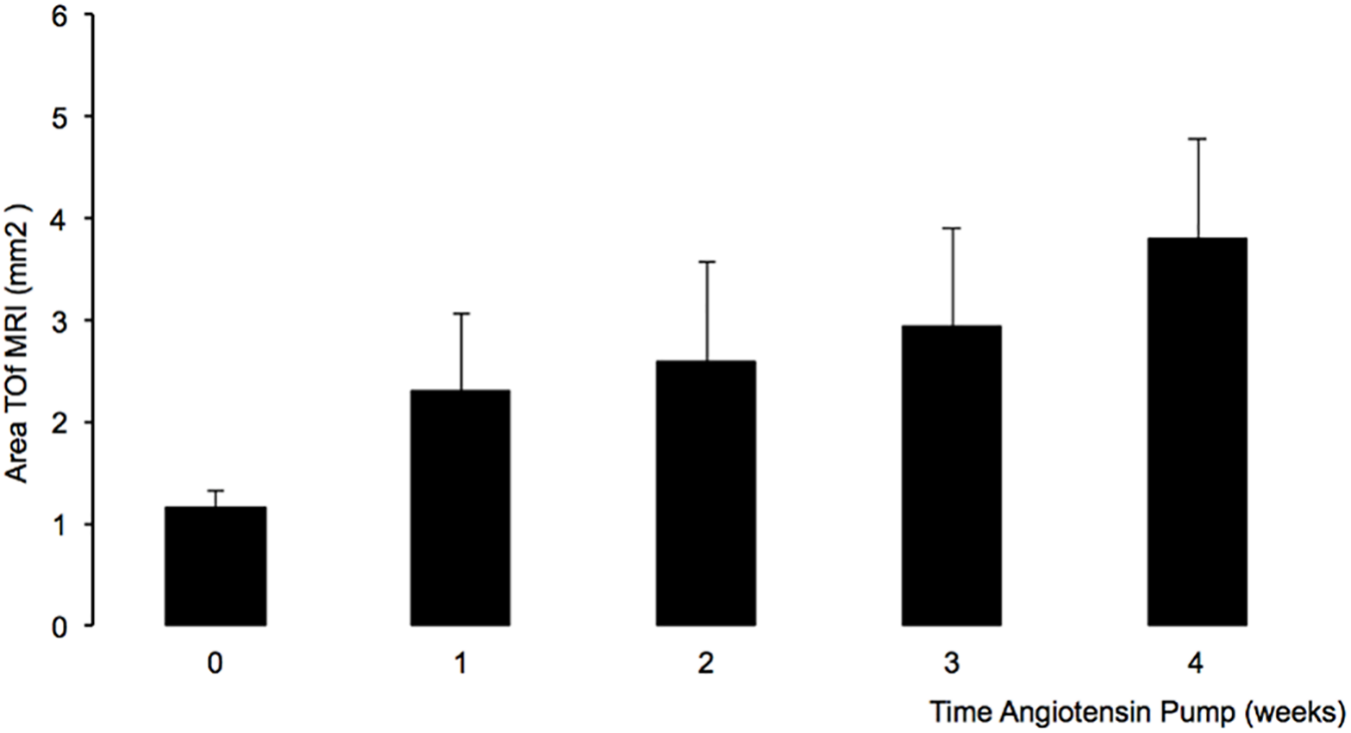
Time course of aortic dilatation assessed by MR-angiography on a clinical MR system. *In vivo* assessment of the dimensions of abdominal aortic aneurysm measured in the TOF angiography in an ApoE-/- mouse model of aortic aneurysms. The luminal aortic areas were measured *in vivo* after one, two, three and four weeks of angiotensin II infusion. In the control group (sham group) an average aortic area of 1.16 ± 0.12 mm^2^ was measured. After one week of angiotensin II infusion an average area of 2.3 ± 0.7 mm^2^ was measured. A further dilation was observed after three and four weeks with an average aortic area of 2.94 ± 0.8 mm^2^ and 3.79 ± 1.12 mm^2^. ToF: Time of flight.

The continuous angiotensin II infusion in ApoE-/- mice resulted in the development and progression of abdominal aortic aneurysms. In the sham group the development of abdominal aortic aneurysms was not observed. Using the clinical 3T MRI system the aortic dilation at different stages of aneurysm development could be clearly visualized. In the sham group an average aortic area of 1.16 ± 0.12 mm^2^ was measured (Fig 3). After one week of angiotensin II infusion an average area of 2.3 ± 0.7 mm^2^ was measured. A further dilation was observed after three and four weeks with an average aortic area of 2.94 ± 0.8 mm^2^ and 3.79 ± 1.12 mm^2^ (Fig 3).

### Comparison of signal to noise measurements of magnetic resonance angiography with preclinical high-frequency ultrasound system

**Fig 4:**
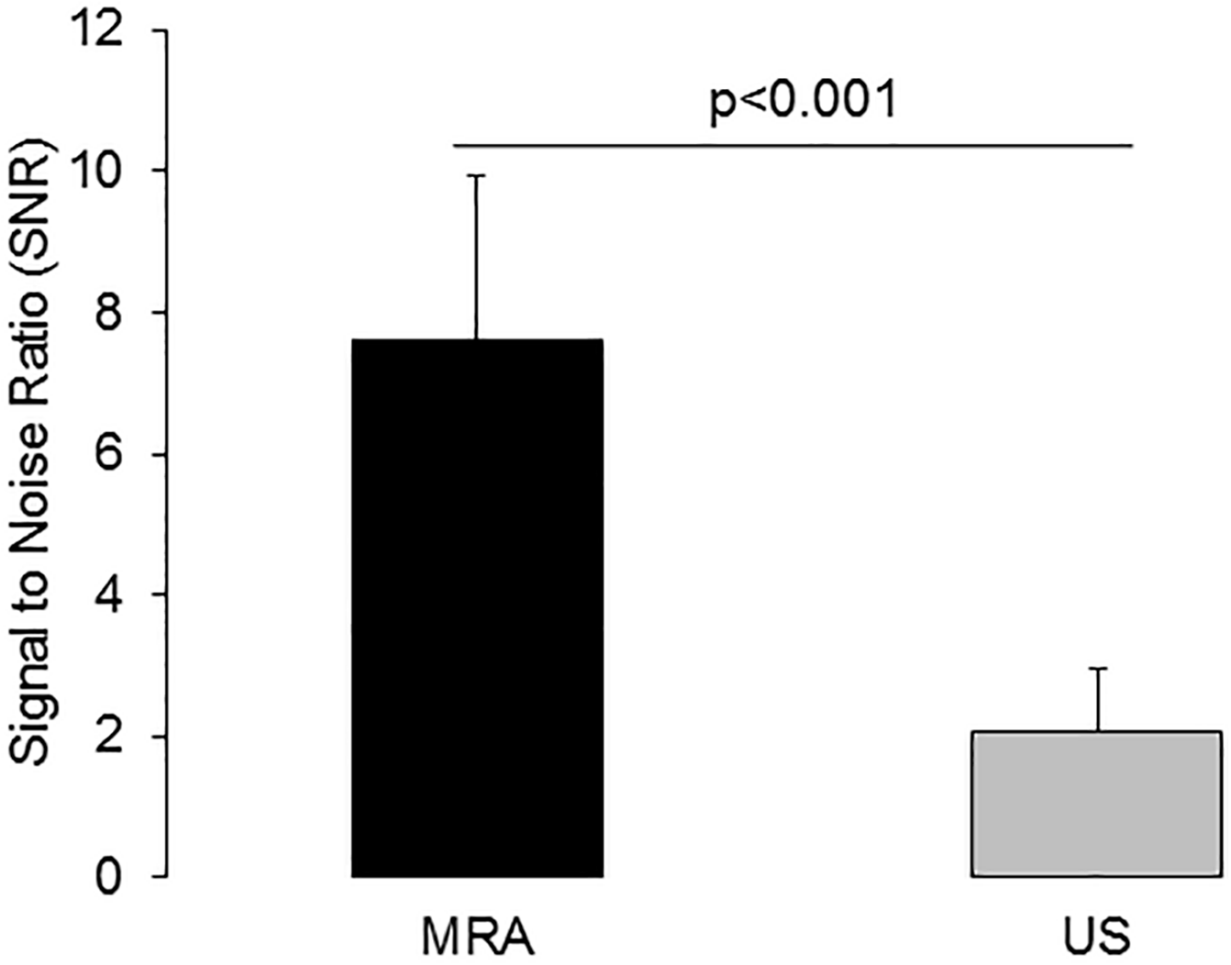
Signal to noise measurements of the aortic lumen on a clinical MR system and on a dedicated high-frequency ultrasound system. This figure shows that magnetic resonance angiography (MRA, black bar) demonstrated a significantly (p < 0.001) higher signal to noise ratio (SNR) compared to ultrasound (US, grey bar). MRI and ultrasound measurements were performed at comparable locations of the aorta. The time-of-flight technique in MR and the B-mode in ultrasound are techniques which are also frequently used in a clinical setting.

In clinical practice, the luminal area/diameter is assessed based on a TOF angiogram or a contrast enhanced angiogram in MRI. In ultrasound diameter measurements are usually performed in the B-mode. Therefore, we used the according images in our study for signal measurements. Signal to noise measurements (SNRs) were performed in the same animal at the same location in both the MR angiogram and ultrasound images (Fig 4). Signal to noise measurements yield a significantly (p < 0.001) higher values for MR-angiography compared to ultrasound images (7.6 ± 2.4 versus 2.1 ± 0.9).

### Comparison of lumen measurements of magnetic resonance angiography and preclinical high-frequency ultrasound system with histopathology

**Fig 5:**
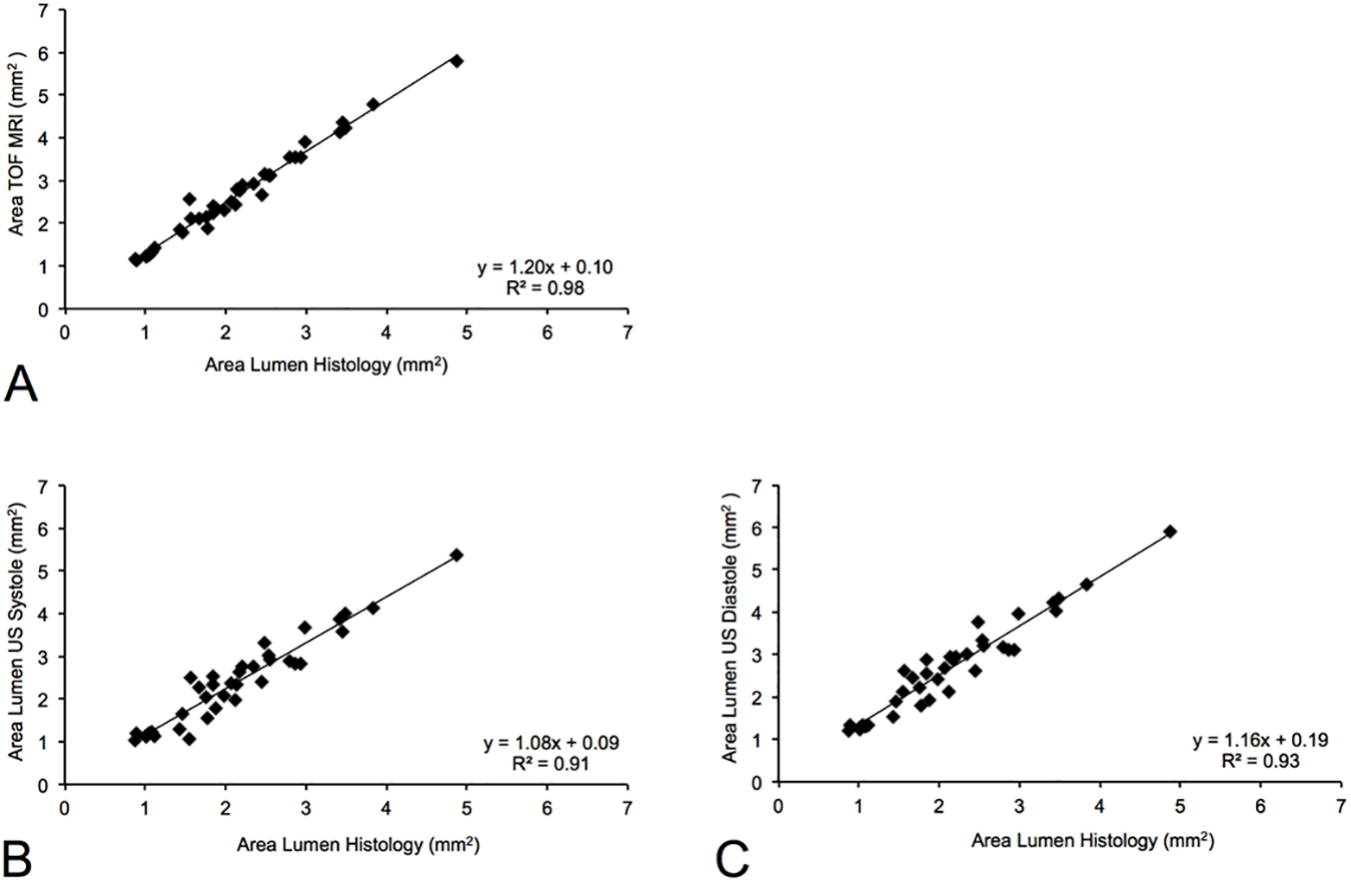
*In vivo* area measurements on the TOF MR-angiography and dedicated high-frequency ultrasound compared to *ex vivo* histopathology. The closest correlation (A) between *in vivo* measurements and *ex vivo* histology was found for the TOF angiogram (R^2^ = 0.98; p < 0.001). This can be explained by the higher signal to noise ratios of MRA compared to ultrasound. A high, however slightly lower however significant correlation (B, C) was measured for ultrasound in systole (R^2^ = 0.92; p < 0.001) and diastole (R^2^ = 0.93; p < 0.001). A slight overestimation of luminal areas was measured for both MR-angiography and ultrasound. This can be explained with the shrinkage of the histological specimens following the processing of the tissue samples. MRA: Magnetic resonance angiography.

Both the MR-angiography acquired on a clinical MRI system and the dedicated preclinical high-frequency ultrasound system enabled a reliable differentiation of the different stages of aortic aneurysms. All *in vivo* measurements were correlated with measurements on *ex vivo* histology (Elastica-van-Giesson stain, reference standard). Area measurements on *in vivo* MR angiograms showed the closest correlation with *ex vivo* measurements (R^2^ = 0.98; p < 0.001, Table 1), while *in vivo* measurements slightly and systemically overestimated the size of the aneurysmal area (Fig 5). This can be explained by the shrinkage of the histological specimens following the processing of the tissue samples. In high-frequency ultrasound, systolic and diastolic area measurements showed a strong, however slightly lower, correlation with *ex vivo* histology compared to the MR-angiography. The correlation coefficient for systolic area measurements was R^2^ = 0.91 (p < 0.001) and for diastolic area measurements R^2^ = 0.93 (p < 0.001). Comparable to MR measurements, ultrasound measurements in both cardiac phases resulted in a slight systemic overestimation of the area of the aortic aneurysm. This can be explained by the shrinkage of the histological specimens following tissue processing.

**Table 1:** Summary of results from in vivo magnetic resonance imaging, ultrasound and ex vivo histology.

	Mean	SD	95% CI			R^2^	p value
**MRI vs US systole**	-0.26	0.34	-0.94	to	0.42	0.91	<0.001
**MRI vs US diastole**	0.03	0.26	-0.49	to	0.54	0.94	<0.001
**MRI vs Histology**	-0.50	0.26	-1.01	to	0.01	0.98	<0.001
**US systole vs Histology**	-0.25	0.31	-0.86	to	0.37	0.91	<0.001
**US diastole vs Histology**	-0.53	0.33	-1.20	to	0.14	0.93	<0.001
**Interobserver MRI**	0.04	0.21	-0.37	to	0.46	0.96	<0.001
**Interobserver US**	0.05	0.42	-0.78	to	0.88	0.87	<0.001

This table summarizes the results from the different in vivo imaging modalities (MRI, ultrasound in systole and diastole, including interobserver variability) and ex vivo histology. Regarding in vivo measurements, the closest correlation was found between MRI and ultrasound measurements in diastole. MRI showed the closest correlation with area measurements on ex vivo histology. Interobserver variation was smaller for MRI compared to ultrasound. 95% CI: 95% confidence interval.

### Comparison of lumen measurements of magnetic resonance angiography with high-frequency ultrasound system

**Fig 6:**
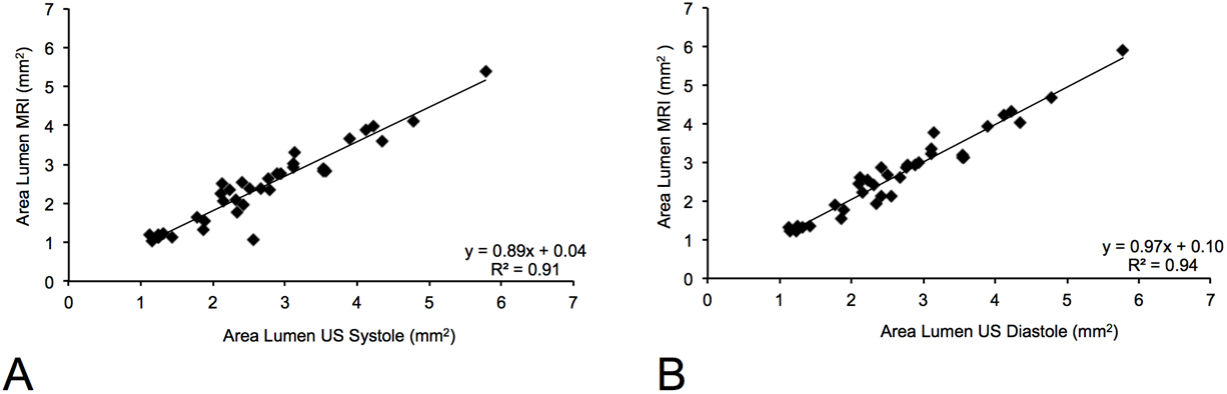
Correlation of area measurements on the TOF MR-angiography with a dedicated high-frequency ultrasound. Area measurements of aortic aneurysms showed a close correlation between time-of-flight MR angiography and high-frequency ultrasound measurements in both cardiac phases. Measurements in diastole (R^2^ = 0.94; p < 0.001) showed a closer correlation compared to measurements in systole (R^2^ = 0.91; p < 0.001). This can be explained by the acquisition technique of the time-of-flight angiography. The time-of-flight angiography is acquired continuously through both cardiac cycles. The resulting images therefore reflect the larger diameter in diastole.

Luminal area measurements on TOF MR-angiography showed a close correlation with measurements derived from the dedicated preclinical high-frequency ultrasound system in systole and diastole (Fig 6). However, the correlation was slightly higher in diastole (R^2^ = 0.94; p < 0.001) compared to the systole (R^2^ = 0.91; p < 0.001). As the TOF angiography continuously acquires images throughout systole and diastole, the resulting image reflects the larger diameter acquired in diastole, as summation effects occur. Therefore, a slightly better correlation was found between TOF angiography and ultrasound images acquired in diastole.

### Interobserver agreements magnetic resonance angiography and ultrasound measurements

**Fig 7:**
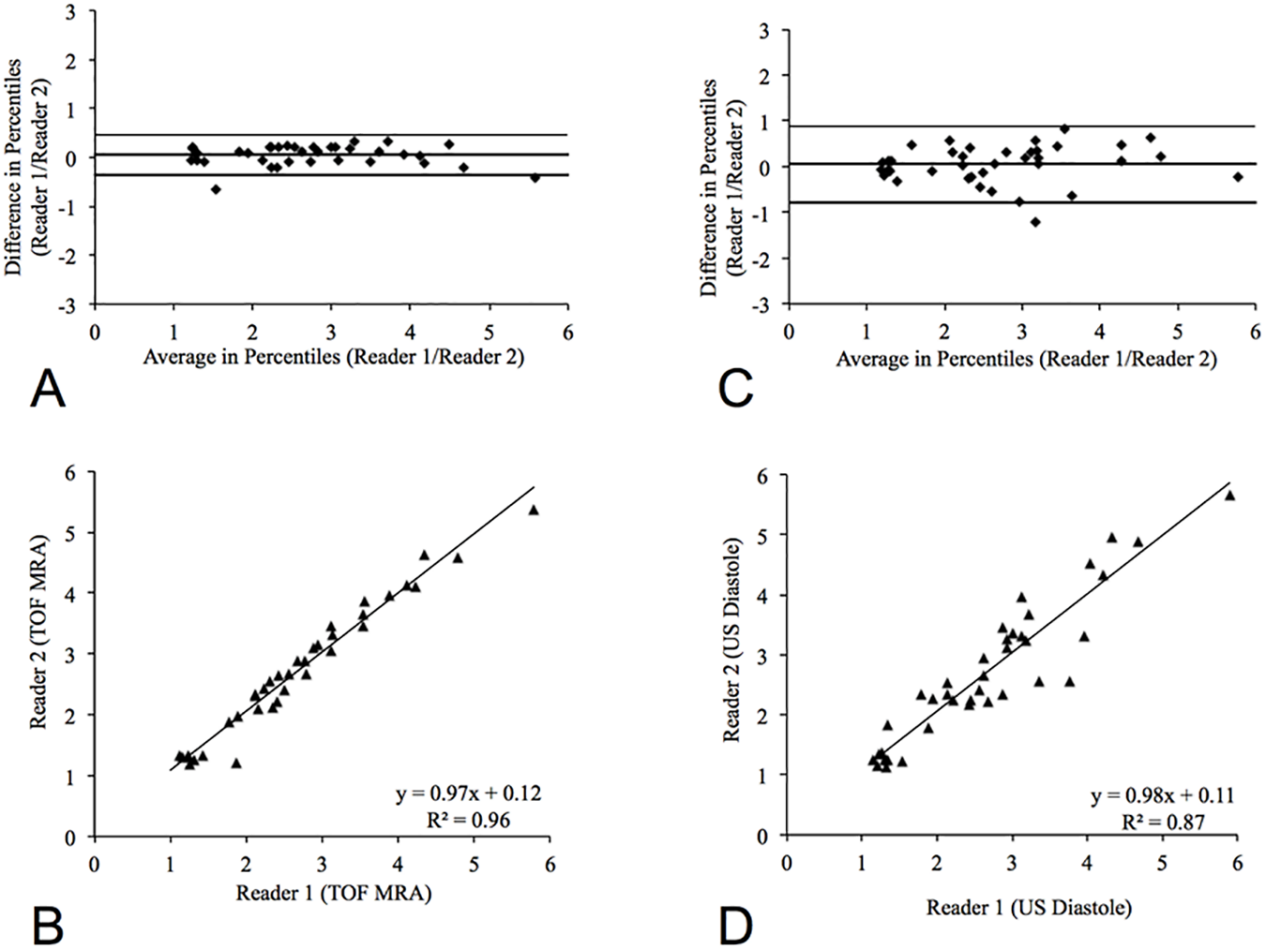
Reproducibility of MR-angiography and ultrasound measurements. MR-angiography (A, B) measurements showed the highest reproducibility of measurements between both readers with the smallest 95% confidence intervals (-0.73 to 0.46, R^2^ = 0.96; p < 0.001). Intraobserver reproducibility for high-frequency ultrasound was slightly lower with slightly wider 95% confidence intervals (-0.7 to 0.88, R^2^ = 0.87; p < 0.001). This can be explained by the overall higher signal to noise ratio of the magnetic resonance angiography and therefore the improved delineation of the vascular boundaries.

Interobserver correlation for area measurements in MR-angiography showed a close correlation between both image readers (R^2^ = 0.96; p < 0.001) (Fig 7). The associated 95% confidence interval (CI) for the correlation range was -0.73 to 0.46. Interobserver correlation for area measurements for high-frequency ultrasound also showed a strong correlation between both readers (R^2^ = 0.87; y = 0.98x + 0.11; p < 0.001). The associated 95% confidence interval (CI) for the correlation range was 0.88 to -0.78. The interobserver correlation for MR-angiography measurements was slightly higher compared to the interobserver correlation for high-frequency ultrasound measurements (Fig 7).

## Discussion

In this imaging study, we report that MR-angiography (MRA), performed on a clinical 3T MR scanner, enables the reliable detection and quantification of the different stages of aneurysm development in an experimental mouse model.

### Imaging techniques for the assessment of aortic aneurysms

Different imaging modalities such as MRI and ultrasound can be used for the assessment of the size of the aortic lumen [9]. In MRI, different techniques can be used for the visualization of vessels. These include non-contrast enhanced and contrast-enhanced techniques [9]. In an experimental setting with small animals, such as mice, non-contrast enhanced techniques have several advantages. The main advantage is that imaging can be repeated at limitless timepoints longitudinally in a single animal. A further advantage is, that TOF imaging sequences do not rely on a first pass of the contrast agent. Furthermore, if a study with e.g. a specific or unspecific MR probe or contrast agent is performed, no additional contrast agent for the angiography is required. Such an additional administration of contrast agent could e.g. interfere with the biokinetics of the evaluated MR probe. The used TOF MR imaging technique is based on the use of relatively short MR repetition times [23]. At each acquisition step, fresh or unsaturated nuclear spins flow into the imaging slice driven by the arterial blood stream. This leads to the bright signal in this MR angiographic technique. In the clinical setting, this imaging technique is routinely used for the visualization of e.g. the cerebral arteries [23]. In preclinical models, this technique is especially useful for the assessment of the vascular system, as it is a relatively fast imaging sequence enabling high throughput preclinical serial imaging, e.g. for pharmacological studies.

A further technique which is used in the preclinical and clinical setting is ultrasound of the aorta. Ultrasound has the advantage that it enables image acquisition with a high temporal resolution which goes beyond the temporal resolution of standard MR imaging sequences. In this study, ultrasound images could therefore be analyzed in end-diastole and end-systole. Various clinical imaging studies have shown that ultrasound is associated with potential limitations [24]. This imaging technique is dependent on the level of experience the operator has. Additionally, image quality depends on the acoustic window. Compared to MR imaging, the luminal boundaries are more challenging to delineate on conventional ultrasound images compared to MR-angiography. This preclinical study confirmed what clinical studies have suggested, the interobserver reproducibility is higher in MR measurements compared to ultrasound measurements [9].

Besides magnetic resonance imaging and ultrasound, computed tomography (CT) can also be used for the evaluation of aortic aneurysms. The main advantage of computed tomography is that image acquisition is relatively fast compared to MRI and ultrasound. The acquisition of the complete aorta is usually performed with in one minute. The main disadvantage of CT however is, that it relies on the use of ionizing radiation. This is especially important in the context of frequent follow up examinations, which are usually required for the evaluation of aortic aneurysms. Additionally, a CT angiography is in clinical practice performed in combination with iodinated contrast agents. In contrast, MRI and ultrasound enable the visualization of the aorta without the need for contrast agents or ionizing radiation.

In summary, in this study we could demonstrate that MR-angiography in an experimental mouse model can reliably be performed on a clinical 3T MR system. In vivo area measurements derived from MRI showed a good correlation with measurements from high-frequency ultrasound and histopathology. Area measurements in MR slightly overestimated the luminal area, compared to histopathology. This can be explained by the tissue shrinkage resulting from the processing of histological samples. The interobserver variability of MR measurements was lower compared to ultrasound measurements. This could be explained by the 3D MR acquisition. This is of relevance, as a high interobserver reproducibility is important, especially for follow-up measurements during the development of aortic aneurysms. To the best of our knowledge, this is the first study that reports the reliable assessment of aortic aneurysms in a mouse model using a clinical 3T system with a close correlation to ultrasound and histological area measurements.

### Relevance of experimental models in the context of aortic aneurysms

In the group of cardiovascular diseases, the 3rd most common cause of sudden death is the rupture of aortic aneurysms. Overall the pathophysiology of the development of aortic aneurysms is not fully understood yet [25]. This is reflected by the fact that most aortic aneurysms are still classified as nonspecific [26]. Regardless of the underlying reason, aortic aneurysms are recognized by a progressive dilation of the aortic lumen. If this process is not detected, progressive dilation can lead to aortic rupture with potentially deadly consequences for the patient. The degree of dilation of the aorta is currently the best established parameter to assess the risk of rupture [27, 28]. To further develop our understanding of the etiology of the onset, development and progression of aortic aneurysms different animal models have been used in previous studies [29–31]. Both pharmacological and surgical techniques have been applied to induce the reproducible development of aortic aneurysms [32]. The most widely used model is based on an ApoE-/- mouse in which angiotensin II is continuously administered [17]. This leads to the spontaneous, reliable and reproducible onset and progression of aortic aneurysms without the need for a direct surgical manipulation of the aorta. For an accurate and reproducible assessment of aneurysm development, measurements on histopathological slides are usually performed *ex vivo*. The drawback of such an *ex vivo* approach is the high number of animals required to achieve a sufficient statistical power.

MR angiography performed on a clinical 3T MR scanner could give more research groups access to a reliable in vivo MR imaging technique for the investigation of aortic aneurysms in experimental models. Additionally, based on in vivo MR imaging, the number of research animals required for the investigation of the different stages of aortic aneurysms could be reduced.

## Limitations

Clinical MRI scanners with a clinically used field strength (1.5–3.0 T) yield a lower signal-to-noise ratio (SNR) compared scanner with a high field strength (4.7–16.4 T), we however did not perform a comparison between ultrahigh field dedicated preclinical MR imaging systems and clinical MR systems. We did not perform Doppler color velocity ultrasound imaging as it was not available on our ultrasound system. However, area measurements in ultrasound are in clinical practice usually performed on standard B-mode images, as they offer the highest spatial resolution.

## Conclusion

This study demonstrates that MR-angiography performed on a clinical 3T MR scanner enables the reliable detection and quantification of aortic dilatation at the different stages of aneurysm development in an experimental mouse model. Interobserver analysis demonstrated a higher reproducibility of MR measurements compared to ultrasound measurements. Both MR-angiography and ultrasound showed a close correlation with histology.
